# Microbial taxa in dust and excreta associated with the productive performance of commercial meat chicken flocks

**DOI:** 10.1186/s42523-021-00127-y

**Published:** 2021-10-02

**Authors:** Yugal Raj Bindari, Robert J. Moore, Thi Thu Hao Van, Stephen W. Walkden-Brown, Priscilla F. Gerber

**Affiliations:** 1 Animal Science, School of Environmental and Rural Science, University of New England, Armidale, NSW 2351 Australia; 2 School of Science, RMIT University, Bundoora West Campus, Plenty Rd, Bundoora, VIC 3083 Australia

**Keywords:** Chicken, Microbiota, Performance, Signatures, Dust, Excreta

## Abstract

**Background:**

A major focus of research on the gut microbiota of poultry has been to define signatures of a healthy gut and identify microbiota components that correlate with feed conversion. However, there is a high variation in individual gut microbiota profiles and their association with performance. Population level samples such as dust and pooled excreta could be useful to investigate bacterial signatures associated with productivity at the flock-level. This study was designed to investigate the bacterial signatures of high and low-performing commercial meat chicken farms in dust and pooled excreta samples. Poultry house dust and fresh pooled excreta were collected at days 7, 14, 21, 28 and 35 of age from 8 farms of two Australian integrator companies and 389 samples assessed by 16S ribosomal RNA gene amplicon sequencing. The farms were ranked as low (n = 4) or high performers (n = 4) based on feed conversion rate corrected by body weight.

**Results:**

Permutational analysis of variance based on Bray–Curtis dissimilarities using abundance data for bacterial community structure results showed that company explained the highest variation in the bacterial community structure in excreta (R^2^ = 0.21, *p* = 0.001) while age explained the highest variation in the bacterial community structure in dust (R^2^ = 0.13, *p* = 0.001). Farm performance explained the least variation in the bacterial community structure in both dust (R^2^ = 0.03, *p* = 0.001) and excreta (R^2^ = 0.01, *p* = 0.001) samples. However, specific bacterial taxa were found to be associated with high and low performance in both dust and excreta. The bacteria taxa associated with high-performing farms in dust or excreta found in this study were *Enterococcus* and *Candidatus Arthromitus* whereas bacterial taxa associated with low-performing farms included *Nocardia*, *Lapillococcus*, *Brachybacterium*, *Ruania*, *Dietzia*, *Brevibacterium*, *Jeotgalicoccus, Corynebacterium* and *Aerococcus.*

**Conclusions:**

Dust and excreta could be useful for investigating bacterial signatures associated with high and low performance in commercial poultry farms*.* Further studies on a larger number of farms are needed to determine if the bacterial signatures found in this study are reproducible.

**Supplementary Information:**

The online version contains supplementary material available at 10.1186/s42523-021-00127-y.

## Introduction

The taxonomic composition of gut microbiota of poultry is affected by various factors such as diet, environment, breed, infectious agents, and management, and has been shown to influence bird health, nutrition, and physiology [1–4]. Methods for monitoring of gut health, which can be defined as the ‘state of symbiotic equilibrium between the microbiota and intestinal tract where animal health and welfare remains unaltered due to dysfunctional intestine’ is a prime focus in poultry research as a healthy gut is required to ensure bird welfare and to prevent production losses [5]. Several experimental studies have shown that differentially abundant bacterial taxa identified in excreta, ileum, and caecum contents, can be correlated with either good or poor bird production performance [6–8]. Specifically, bacterial groups that are commonly known as butyrate producers and resistant starch degraders are usually associated with increased bird feed efficiency [8, 9]. However, the bacterial composition of the gut microbiota of individual birds is highly variable within and between studies and specific bacterial signatures that reliably correlate with good or poor flock performance remain elusive [9, 10].

Within the chicken gut, the highest microbial density and the most complex microbial community is found in the caecum, and therefore caecal content has been commonly used to investigate the gut microbiota [8]. The collection of caecum or gut contents has some drawbacks as it requires birds to be euthanized and precludes longitudinal studies in the same birds [11]. Therefore, there is a need for the development of methods that use non-invasive samples to evaluate gut microbiota [11, 12]. Previous studies evaluating excreta, boot sock samples and caecal droppings have suggested that boot sock samples and caecal droppings could be useful alternatives to study caecal microbiota composition longitudinally [11, 13]. The rationale for the use of these alternative sample types is that they are derived from the gastrointestinal tract of chickens and could therefore be used to infer gut health and perhaps performance. However, the relationship of the microbiota composition using non-invasive population level samples and flock performance is not well understood. As measurements of productive performance and management interventions in commercial meat chicken farms are mostly applied at farm-level, a monitoring strategy at a population-level would perhaps be advantageous compared to sampling of individual birds. The use of non-invasive population level samples such as poultry dust, pooled excreta, and litter has several advantages compared to individual sampling of birds, such as ease of sample collection and the use of a small number of samples to represent a population. Dust samples, in particular, are dry, stable and can be shipped at room temperature. A recent study has shown that genomic material of RNA and DNA viruses was stable in poultry dust for at least to 4 months when stored at temperatures up to 37 °C [14].

In this study, poultry house dust and fresh pooled excreta from the floor were collected weekly from eight commercial meat chicken farms of two Australian integrator companies. The farms were ranked as low or high performers based on feed conversion ratio corrected for body weight by the integrator companies. We hypothesized that microbiota profiles of pooled excreta and dust collected from poultry farms would provide specific bacterial signatures associated with high and low-productive farm performance. This study was specifically designed to (1) Determine and compare the bacterial taxa of poultry dust and pooled excreta in the two integrator companies; (2) Determine if the bacterial taxa in dust and pooled excreta could differentiate high and low-performing farms.

## Methodology

### Farms and samples collection

The study was conducted on 16 flocks from eight commercial meat chicken farms (n = 2 flocks/farm) in a 2 × 2 × 5 factorial arrangement with two Australian integrator companies (A and B), two levels of farm productive performance (high and low), and five sampling times (7, 14, 21, 28 and 35 days of chicken age) as previously described [15]. Details of the farms are included in Additional file 1. Samples were collected between August 26 and November 19, 2019. The integrator companies varied in a number of management practices, including the choice of chicken strain, geographic location, and feed formulation. All farms from company A were located in the outskirts of Sydney in New South Wales, used the Cobb strain of chickens and used a range of bedding materials (wood shavings, sawdust, biobedding) (Additional file 1). All farms from company B were located in the outskirts of Adelaide, South Australia, used the Ross strain of chickens and straw as bedding material. Two farms with consistent high performance and two farms with consistent low performance based on historical production data were selected by veterinarians from each company. The productive performance ranking of each farm, which is based on the corrected feed conversion rate for the whole farm, was then recorded at the end of the production cycle which confirmed that the studied flocks ranked high (above the 50 percentile of production) or low (below the 50 percentile). Pooled excreta and dust samples were collected weekly from two poultry houses in each farm at 7, 14, 21, 28 and 35 days of the production cycle. Settled dust samples were collected using two funnels held in an apparatus suspended at 1.5 m height on the wires in the house that support feeders and waterers. The funnels which were put in place on the day of chick placement captured setting dust and directed it into a collection vial attached to the funnel that was removed at each sampling and replaced with a new vial. Settled dust samples were dry and with fine consistency and submitted vials had enough material for testing (at least 10 mg of dust). Five fresh individual excreta samples were collected from the poultry house floor to form one pooled sample and four pooled excreta samples were collected from each poultry house in a stratified sampling pattern. A total of 406 samples (dust = 138, excreta = 268) were received from the participant farms, from which 17 samples were discarded because of poor quality DNA making a total of 389 samples analysed in this study (dust = 131; excreta = 258).

### DNA extraction

Previous experience with these types of samples had shown that no one DNA extraction method gave good DNA yields from both dust and excreta. To maximise DNA quality and yields from each sample type different extraction kits were used. DNA extraction of dust was performed using the QIAamp® Fast DNA Stool Mini Kit (Qiagen, Hilden, Germany) according to the manufacturer’s instructions with minor modifications [15]. Briefly, 0.4 g of 1 mm glass beads were added to 2 ml microtubes containing 1 ml of InhibitEX and 10 mg of dust and homogenised for 5 min at maximum speed using a Qiagen Tissue Lyser II (Qiagen, Hilden, Germany). The homogenised suspensions were then heated at 95 °C for 10 min. DNA extraction from excreta was performed using the DNeasy PowerSoil Pro Kit (Qiagen, Hilden, Germany) according to the manufacturer’s instructions, except that 50 mg of excreta were homogenised for 5 min with PowerBead at maximum speed using Qiagen Tissue Lyser II (Qiagen, Hilden, Germany) with subsequent heating of homogenised suspensions at 90 °C for 10 min. It is acknowledged that different extraction methods can result in different 16S amplicon analysis results [16]. The type of sample and DNA extraction kits affect the quality of DNA and composition of bacteria following 16S rRNA gene sequencing using Illumina MiSeq sequencer [16, 17] but such differences are mainly restricted to the yield of Gram-positive bacterial DNA due to efficiency of cell breakage. Both methods used in the current study used efficient bead-beating protocols to minimise this effect.

### 16S rRNA gene amplification and analysis

Microbiota composition was assessed by sequencing of amplicons across the V3-V4 region of 16S rRNA genes. Amplicons were produced using custom-designed barcoded primers targeting the 343–806 region, ACTCCTACGGGAGGCAGCAG (forward primer) and GGACTACHVGGGTWTCTAAT (reverse primer), primers also contained spacer sequences and Illumina sequencing linkers, following the design of Fadrosh et al. [18]. The amplicons were sequenced on an Illumina MiSeq Sequencer using 2 × 300 bp paired-end reads. Sequence data were trimmed with Trimmomatic and then fatsq files were analysed using DADA2 in QIIME2 v2020.6 [19] to denoise and produce Amplicon Sequence Variants (ASVs). ASVs were clustered at 99% identity using the VSEARCH plugin [20]. Taxonomy was assigned using the SILVA database. All of the downstream statistical microbial data analysis and visualisation were done using Calypso software [21]. A total of 9624 ASVs were found. The sequence data used for analysis is available in NCBI under BioProject accession number PRJNA730489.

### Statistical analysis

The bacterial community composition data was normalised using Hellinger transformation before statistical comparison. Statistical analyses were performed using the Calypso software (http://cgenome.net/wiki/index.php/Calypso) except for the permutational multivariate analysis of variance (PERMANOVA) which was performed using the PRIMER v7 software including the PERMANOVA + add-on module [22].

Distance based redundancy analysis (dbRDA) using Bray–Curtis dissimilarity was used to visualize the difference in bacterial community structure between companies in dust and excreta and principal-coordinate analysis (PCoA) using Bray–Curtis dissimilarity was used to visualize the differences in bacterial community structures between bird age (7, 14, 21, 28 and 35 days). To examine the effects of sample type, company, bird age, performance and their interactions in the bacterial community structures, permutational multivariate analysis of variance (PERMANOVA) based on Bray–Curtis dissimilarities using the default setting of Type III sum of squares was used [23]. Homogeneity of multivariate dispersion was tested using permutational analyses of multivariate dispersions (PERMDISP) [24]. Linear discriminant analysis effect size (LEfSe) was used to test the differences in abundance of the bacterial taxa between companies (A and B). The top 50 most abundant taxa were selected and Wilcoxon test, area under the curve (AUC) and odds ratio (OR) were used to identify bacterial taxa more prevalent in high and low-performing farms. *P* values were corrected with the false discovery rate and adjusted *p* value (q) < 0.05 was considered statistically significant. Bacterial taxa with significantly different (q < 0.05) abundance in high and low-performing farms with an AUC ≥ 0.90 were subjected for further OR analysis. The higher likelihood of bacterial taxa occurring in a certain production performance category (high, low) (AUC ≥ 0.90, Wilcoxon test q value < 0.05) were considered as ‘signatures’ of that production outcome.

## Results

### Company and age explained the most variation in the structure of bacterial community in pooled excreta and dust while performance explained the least variation in both sample types

As indicated in material and methods two different methods were used for DNA extraction of dust and excreta which could add variability on the detected bacterial communities. Therefore, data was analysed by PERMANOVA using model accounting to all variables in the study (sample type, company, age and performance) to assess if the microbial taxa was consistent between samples types and also for each sample type separately.

When analysing the whole model, sample type explained the most variation in the bacterial community structure (12%), followed by company (6%), and bird age (5%), while farm performance explained the least variation (1%) with significant interaction among these factors (Additional files 2; 3).

In dust samples, age explained the most variation in the bacterial community structure (13%), followed by company (11%) and the least variation was explained by performance (3%), with significant interactions among these factors (Table 1). In excreta, company explained most of the variation (21%) followed by bird age (10%) and performance (1%) with significant interactions among these factors (Table 1).

**Table 1:** Permutational multivariate analysis of variance (PERMANOVA) results showing the influence of company, age, performance and their interactions on the bacterial community structure in excreta and dust samples

Sample type	Source	df	Sum of squares	Pseudo-F	R^2^	P value	PERMDISP *P* value
Excreta	Company	1	167,120	91.10	0.21	0.001	< 0.001
	Age	4	81,418	11.10	0.10	0.001	0.008
	Performance	1	10,863	5.92	0.01	0.001	0.03
	Age × company	4	39,511	5.38	0.05	0.001	
	Company × performance	1	12,723	6.94	0.02	0.001	
	Age × performance	4	14,605	1.99	0.02	0.001	
	Performance × age × company	4	19,093	2.60	0.02	0.001	
	Residuals	238					
Dust	Company	1	29,643	21.94	0.11	0.001	0.003
	Age	4	35,962	6.65	0.13	0.001	< 0.001
	Performance	1	9151.6	6.77	0.03	0.001	0.11
	Age × company	4	15,488	2.87	0.06	0.001	
	Company × performance	1	4559.6	3.37	0.02	0.001	
	Age × performance	4	8810.5	1.63	0.03	0.001	
	Performance × age × company	4	11,313	2.09	0.04	0.001	
	Residuals	111					

Df, degrees of freedom; R^2^, proportion of the variance explained by each model; PERMDISP, permutational analyses of multivariate dispersion

#### Sample type

**Fig. 1:**
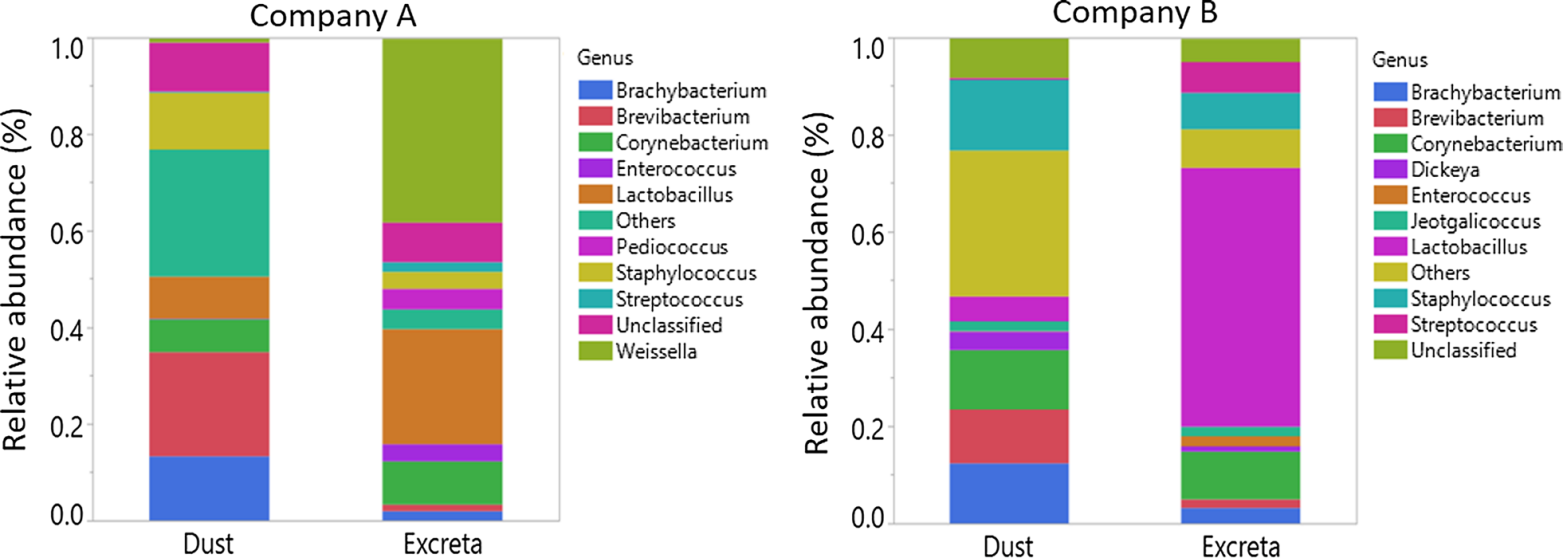
Taxonomic assignment of the bacteria at genus level in dust and excreta stratified by company. The levels of the top 10 most abundant genera were shown. Genera that were not included under top 10 most abundant ones were merged together and presented as ‘Others’

Taxonomic assignment at genus level of the bacteria found in dust and excreta are shown in Fig. 1. The most dominant bacterial genera found in excreta of company A were *Weissella* (mean percentage ± SD 0.38 ± 0.15) and *Lactobacillus* (0.24 ± 0.17) while in company B were *Lactobacillus* (0.53 ± 0.20) and *Corynebacterium* (0.10 ± 0.08) (Fig. 1). In dust samples, *Brevibacterium* (0.22 ± 0.11) and *Brachybacterium* (0.13 ± 0.09) were the most dominant bacterial genera in company A while in company B were *Staphylococcus* (0.15 ± 0.08), *Corynebacterium* (0.12 ± 0.05) and *Brachybacterium* (0.12 ± 0.08) (Fig. 1). At phylum level, the most dominant bacterial phyla in excreta and dust of company A and B were *Firmicutes* (0.38 ± 0.24–0.85 ± 0.13) and *Actinobacteria* (0.14 ± 0.13–0.50 ± 0.24) (Additional file 4). Of the bacterial taxa (genus level) detected in excreta of company A or B, 81% were also detected in dust. The shared bacterial genera between dust and excreta and the bacterial genera that are exclusive to dust or excreta of companies A and B are shown in Additional files 5 and 6.

**Fig. 2:**
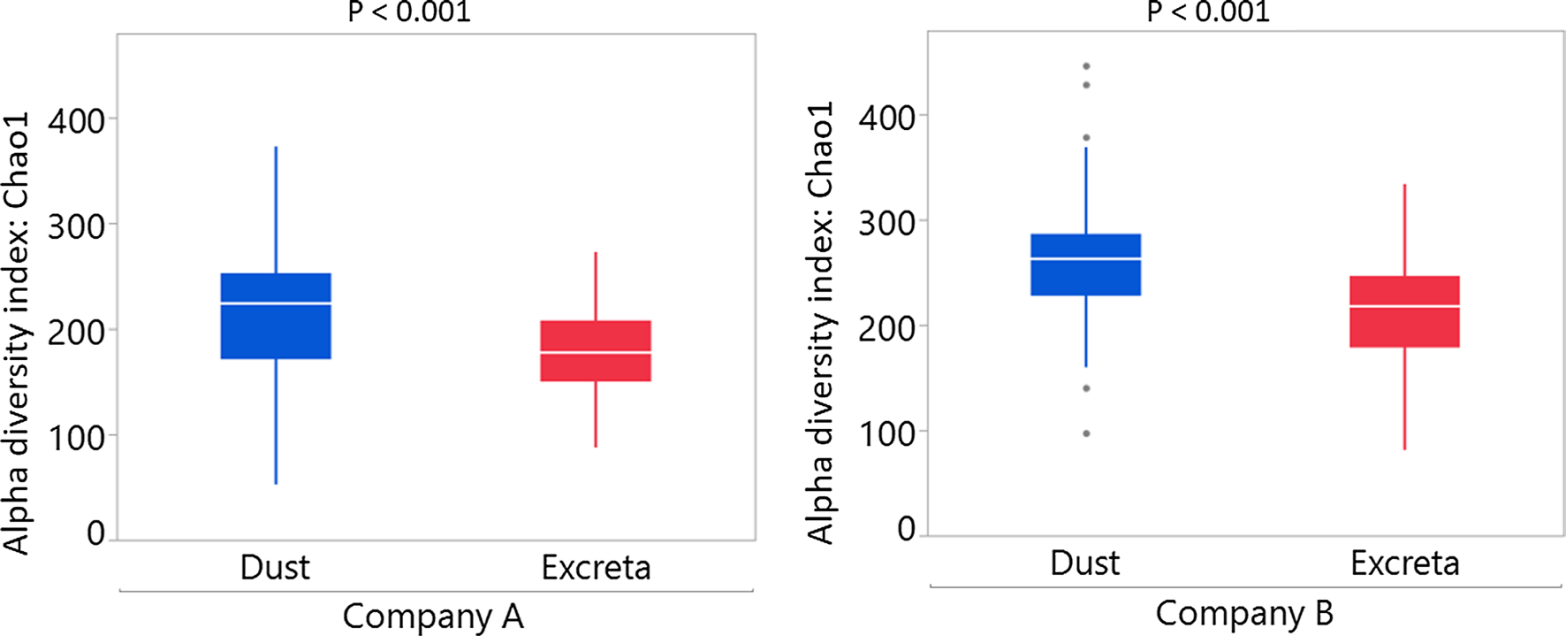
Microbial richness estimated using Chao1 diversity index for dust and excreta of companies A and B. All ages were combined for the analysis

Microbial richness determined by Chao1 diversity index was higher in dust compared to excreta in both companies (*p* < 0.001) (Fig. 2). The bacterial genera that differentiate dust and excreta of companies A and B shown by LEfSe are presented in Additional file 7.

#### Company

**Fig. 3:**
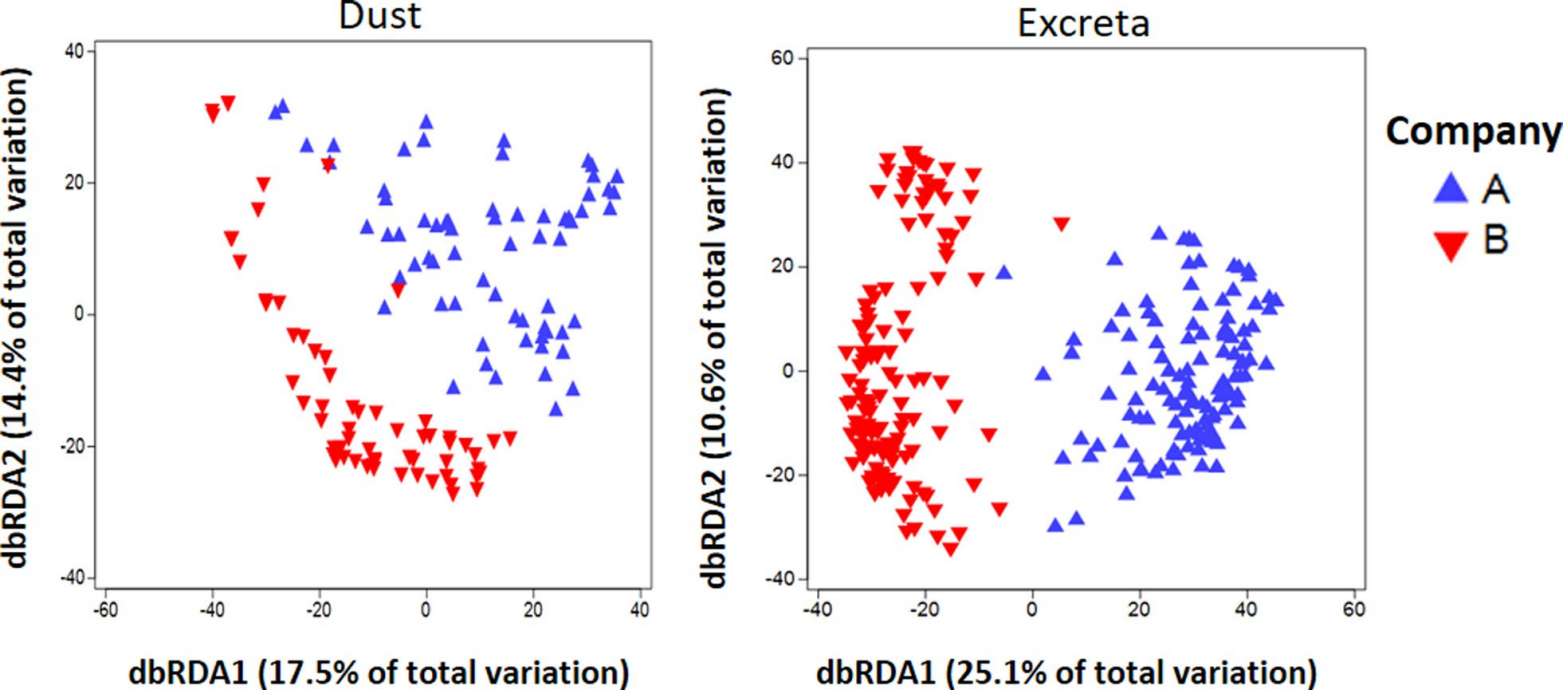
Redundancy analysis showing dissimilarity in the bacterial community structure between companies stratified by sample type

In dust and excreta, 55% and 52% of the bacterial genera were shared between companies, respectively. The shared bacterial genera between companies and the bacterial genera that are exclusive to companies A and B are shown in Additional files 8 and 9. Within each company, microbial communities of pooled excreta (PERMANOVA *p* = 0.001 and R^2^ = 0.23; PERMDISP *p* < 0.001) were more dissimilar than dust (PERMANOVA *p* = 0.001 and R^2^ = 0.12; PERMDISP *p* = 0.003) which was also evident in the redundancy analysis ordination plot (Fig. 3). The bacterial genera that differentiate companies A and B in dust and excreta are shown in Additional file 10.

#### Bird age

As expected, age had a significant effect on the bacterial community structure in both dust and excreta. In excreta, age explained 16% variation in company A (PERMANOVA *p* = 0.001) and 24% in company B (*p* = 0.001) while in dust age explained 19% variation in company A (*p* = 0.001) and 32% in company B (*p* = 0.001).

**Fig. 4:**
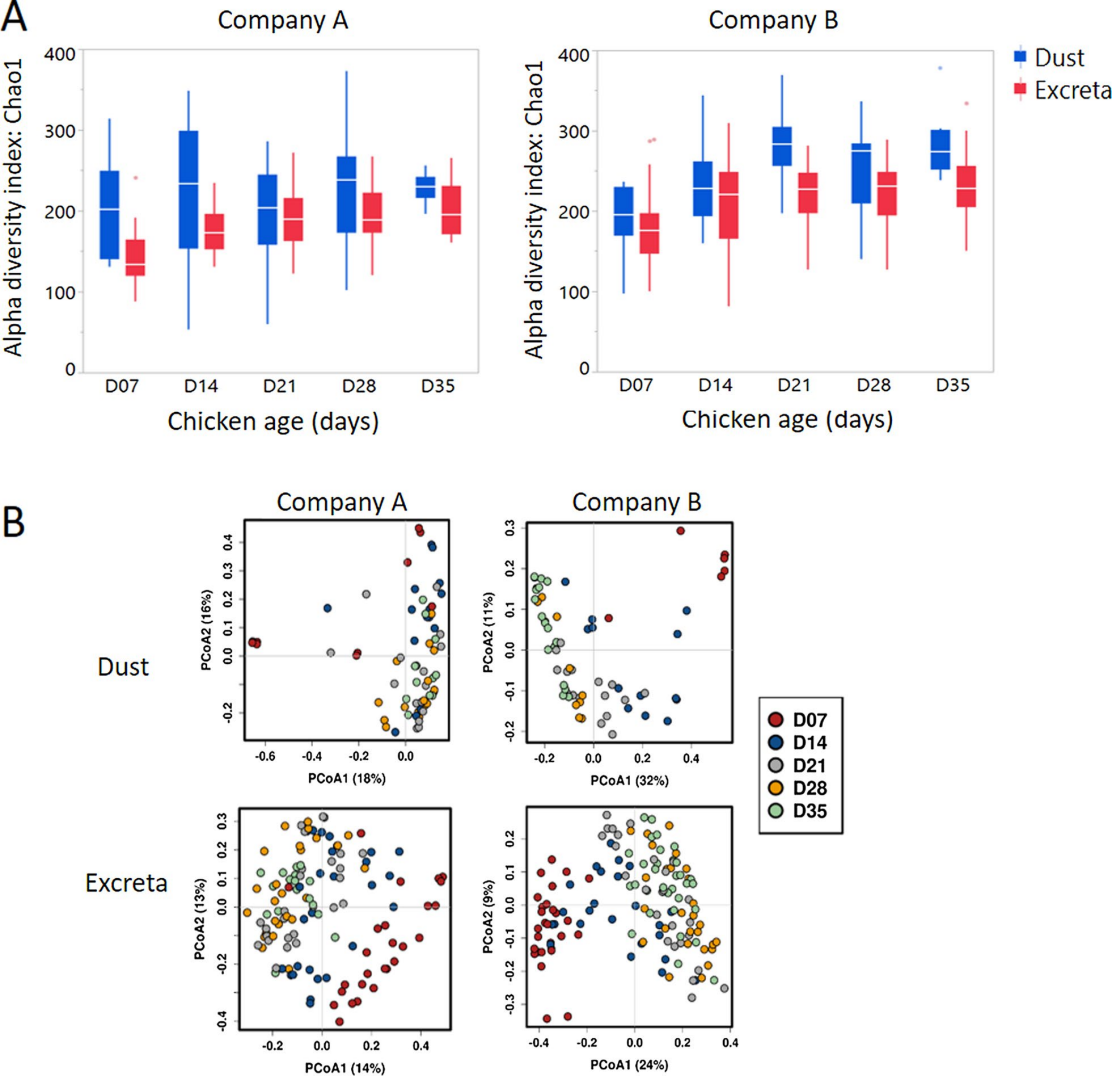
Alpha and beta diversity in dust and excreta samples stratified by company. **a** Alpha diversity was assessed using Chao1 diversity index and **b** principal-coordinate analysis plots using Bray–Curtis dissimilarity showing variation in the bacterial community structure across bird age

**Fig. 5:**
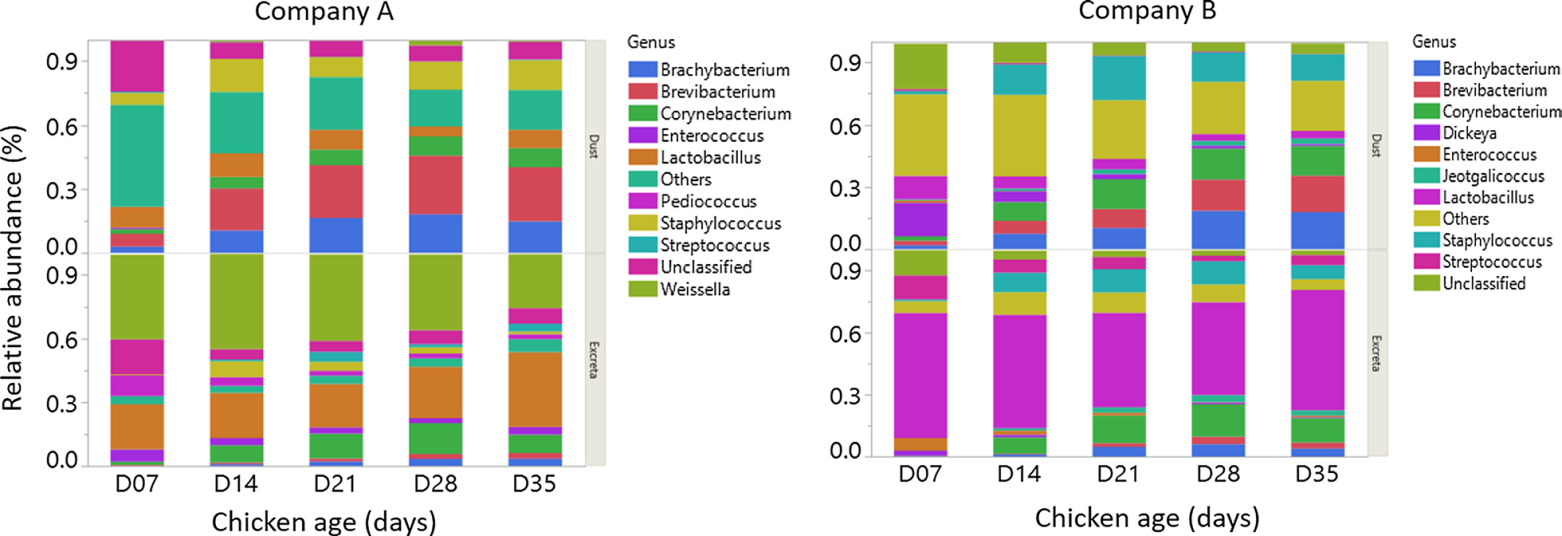
Top 10 most abundant taxa of company A and B farms in dust and excreta samples across different age of the chickens (7, 14, 21, 28 and 35). Genera that were not included under top 10 most abundant ones were merged together and presented as ‘Others’

Bacterial richness was lower at day 7 in company A and B farms in excreta (Fig. 4a) and this age group was the most divergent compared to samples collected afterwards (Fig. 4b) when bacterial richness stabilised. Similar results were seen in dust of company B farms while in company A farms bacterial richness was found to be similar across all ages. Bacterial richness was higher in dust compared to excreta in company A except at days 21 and 28 when dust and excreta had similar microbial richness (Fig. 4a). Similarly, in company B, bacterial richness was higher in dust compared to excreta except at days 7 and 14 when microbial richness between dust and excreta were similar (Fig. 4a). Figure 5 shows the relative abundance of bacterial genera across different ages in dust and excreta samples of company A and B. From days 7–35, excreta were dominated by *Weissella* in company A and *Lactobacillus* in company B*.* Dust was dominated by Unclassified bacterial groups at day 7 and *Brevibacterium* at days 14–35 in company A while in company B dust was dominated by Unclassified bacterial taxa at day 7, *Staphylococcus* at days 14–21, *Corynebacterium* and *Brevibacterium* at days 28 and 35.

#### Performance

Production explained the least variation within the dataset in both dust and excreta (Table 1). PERMANOVA results showed highest variation in the bacterial community structure between high and low production farms in dust at day 7 for company A and at day 28 for company B while in excreta it was at day 7 for company A and day 14 for company B (Table 2).

**Table 2:** Variation in the bacterial community structure between high and low-performing farms of company A and B across the production cycle (7, 14, 21, 28 and 35) in excreta and dust samples using permutational multivariate analysis of variance (PERMANOVA)

Sample type	Company	Chicken Age (days)	PERMANOVA			PERMDISP
			Pseudo-F	R^2^	*P* value	*P* value
Excreta	A	All ages	6.84	0.05	**0.001**	0.33
		7	6.03	0.20	**0.001**	0.90
		14	3.49	0.13	**0.003**	0.87
		21	2.83	0.11	**0.004**	0.65
		28	3.16	0.12	**0.001**	0.16
		35	2.17	0.13	**0.007**	0.81
	B	All ages	3.39	0.02	**0.003**	0.97
		7	1.43	0.05	0.10	0.62
		14	4.67	0.18	**0.001**	0.14
		21	2.01	0.08	0.03	0.19
		28	2.77	0.10	**0.001**	0.36
		35	1.40	0.04	0.10	0.77
Dust	A	All ages	5.54	0.08	**0.001**	**0.02**
		7	2.65	0.25	0.08	0.75
		14	2.83	0.18	**0.001**	0.61
		21	2.78	0.17	**0.002**	0.07
		28	1.97	0.12	**0.04**	**0.004**
		35	2.47	0.22	**0.007**	**0.005**
	B	All ages*	2.97	0.05	**0.01**	0.48
		14	1.78	0.14	0.11	**0.02**
		21	1.66	0.11	0.07	0.21
		28	3.22	0.24	**0.009**	**0.003**
		35	2.11	0.13	**0.02**	0.08

PERMDISP, permutational analyses of multivariate dispersions

*Dust samples collected on day 7 from company B were excluded due to the small sample

Values in bold indicate statistical significance (*p* < 0.05)

### Bacterial signatures of low and high-performing farms

**Fig. 6:**
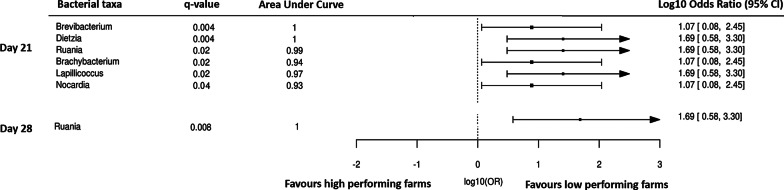
Forest plot of the significantly different bacterial taxa (Wilcoxon q value < 0.05) of company A farms in dust with AUC ≥ 90. The results to the left of the vertical dotted line (line of no effect) denote the increased chance of occurrence of microbiota towards high-performing farm and the results to the right of the vertical dotted line denotes the increased chance of occurrence of microbiota towards the low-performing farm

**Fig. 7:**
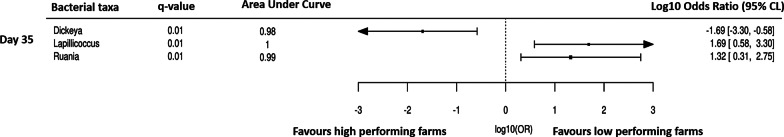
Forest plot of the significantly different bacterial taxa (Wilcoxon q value < 0.05) of company B farms in dust with AUC ≥ 90. The results to the left of the vertical dotted line (line of no effect) denote the increased chance of occurrence of microbiota towards high-performing farm and the results to the right of the vertical dotted line denotes the increased chance of occurrence of microbiota towards low-performing farm

**Fig. 8:**
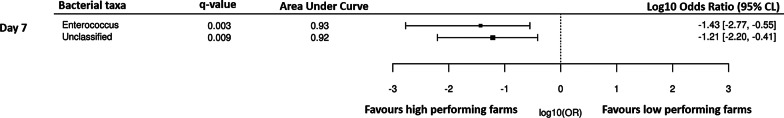
Forest plot of the significantly different bacterial taxa (Wilcoxon q value < 0.05) of company A farms in excreta with AUC ≥ 90. The results to the left of the vertical line (line of no effect) denotes the increased chance of occurrence of microbiota towards high-performing farm and the results to the right of the vertical dotted lines denotes the increased chance of occurrence of microbiota towards the low-performing farm

**Fig. 9:**
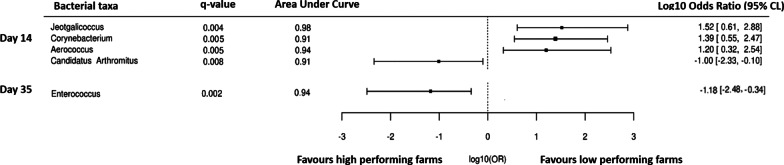
Forest plot of the significantly different bacterial taxa (Wilcoxon q value < 0.05) of company B farms in excreta with AUC ≥ 90. The results to the left of the vertical line (line of no effect) denotes the increased chance of occurrence of microbiota towards high-performing farm and the results to the right of the vertical dotted lines denotes the increased chance of occurrence of microbiota towards the low-performing farm

Although the farm performance explained the least variation in the bacterial community structure, some bacterial taxa were overrepresented in low or high performing farms and met the ‘bacterial signature’ criteria (AUC ≥ 0.90, Wilcoxon test q value < 0.05) (Figs. 6, 7, 8, 9; Additional files 11, 12, 13, 14).

#### Dust samples

No bacterial taxa met the criteria for bacterial signature in high-performing farms of company A; whereas for company B *Dickeya* (day 35) met the bacterial signature criteria (Figs. 6, 7). Although not statistically significant, the abundance of *Dickeya* was also numerically higher in high-performing farms of company A at day 28.

Bacterial taxa in dust of low-performing farms of company A that meet the bacterial signature criteria were *Nocardia* (day 21), *Lapillococcus* (day 21), *Brachybacterium* (day 21), *Ruania* (days 21 and 28), *Dietzia* (day 21) and *Brevibacterium* (day 21); whereas for company B were *Lapillicoccus* (day 35) and *Ruania* (day 35) *(*Figs. 6, 7). Although not statistically significant, the abundance of *Brachybacterium* and *Dietzia* were also observed numerically at a higher level in low-performing farms for company A at days 14 and 28, *Ruania* at days 14, 21 and 35 and *Brevibacterium* at days 7, 14, 28 and 35; whereas in company B the abundance of *Lapillococcus* and *Runia* were numerically higher at days 7 to 28.

#### Excreta

Bacterial taxa that meet the bacterial signature criteria of high-performing farms in company A were *Enterococcus* (day 7) and unclassified group of bacterial taxa (day 7); whereas for company B were *Enterococcus* (day 35) and *Candidatus Arthromitus* (day 14) (Figs. 8, 9). Although not statistically significant, the abundance of unclassified group of bacterial taxa and *Enterococcus* were also observed numerically at a higher level in high-performing farms of company A at days 14–28; while for company B was *Candidatus Arthromitus* at days 7, 21 and 28.

No bacterial taxa meet the bacterial signature criteria of low performing farms for company A; whereas for company B bacterial taxa that meet the bacterial signature criteria were *Jeotgalicoccus* (day 14)*, Corynebacterium* (day 14) and *Aerococcus* (day 14) (Figs. 8, 9). Although not statistically significant, the abundance of *Jeotgalicoccus* was also observed numerically at a higher level in low-performing farms of company B at days 7, 21 and 28, and *Corynebacterium* and *Aerococcus* at days 21 and 28. There was no overlap in the bacterial signatures from high and low-performing farms between dust and excreta of company A and B farms (Figs. 6, 7, 8, 9).

## Discussion

Many studies have associated gut microbiota with feed conversion and weight gain in meat chickens under experimental conditions. We therefore hypothesized that microbiota profiles of population level samples would identify specific bacterial taxa associated with productive performance in commercial meat chicken farms. In the current study, overall farm performance explained the least amount of variation in the bacterial community structure for both dust and excreta samples. Although some bacterial taxa were associated with high or low performance, those signatures were highly dependent on the sample type, company, and the age of the bird.

The microbiota found within the dust of the poultry houses is presumably dependent largely on the excreta and litter microbiota with some influence of the environmental conditions on how those microbiota develop and spread over time. Excreta has been shown to form the major component of dust from turkey houses [25] and broiler chicken houses [26] and Luiken et al. [27] recently showed that poultry dust bacteriome are more diverse but associated with excreta bacteriome composition. Therefore, it is not surprising that 81% of bacterial taxa found in excreta samples were also found in dust. It is possible, however, that the different DNA extraction techniques used in this study could have influenced the detection of bacterial taxa in both sample types.

The microbial community composition varied between companies, with companies A and B sharing 55% of bacterial taxa in dust and 52% in excreta. This was expected as each integrator company used a different chicken breed (Cobb and Ross) and breed has been shown to influence the establishment of gut microbiota [28]. Other factors such as differences in feed formulation, hatchery, housing, bedding material, environment, and management factors within each company are also likely to have driven the observed quantitative differences in the microbial communities [4, 29].

Age also had a significant effect on microbial communities in dust and excreta in both companies, which is similar to previous findings [30–32]. It has been reported that bacterial richness increases during the first weeks of life while the variation in the microbiota of the gut decreases as the age advances [4]. From day 7–35, *Weissella* and *Lactobacillus* were the most dominant taxa in company A and *Lactobacillus* was the most dominant taxon in company B. This is in consistent with previous studies which showed that *Lactobacillus* was the predominant taxon in excreta [12, 33].

Farm performance explained the least variation in the bacterial community structure in both sample types, however, some specific bacterial taxa were differentially abundant in high and low performing farms in both dust and excreta samples. These included *Enterococcus, Dickeya, Candidatus Arthromitus* and an unclassified group of bacterial taxa*.*

*Enterococcus,* which was found as a bacterial signature of high-performing farms in this study, has already been explored for probiotic application in poultry. *Enterococcus* spp. are polysaccharide-degrading bacteria mainly responsible for degradation of mixed linked β-glucan in the intestine of meat chickens [34]. The combination of *Enterococcus* with *Lactobacillus, Bifidobacterium,* and *Streptococcus* spp. have been used as probiotics and shown to have growth-promoting effects comparable to avilamycin treatment [35]. *Candidatus Arthromitus* has been shown to be associated with postnatal maturation of immune function in the mouse gut [36]. The functional roles of genus assigned to unclassified group would need further investigation although this is difficult to perform because of the lack of a system to study their functional role and characteristics [37]. In this study, *Dickeya* spp, which is an economically significant important crop pathogen [38], was also found to be associated with high performing farms. This is probably a coincidental result that needs further investigation.

In this study, no poultry pathogenic bacterial taxa was identified, however, *Nocardia*, *Aerococcus* and *Corynebacterium* found in low-performing farms could potentially cause disease in chickens. Previous research has shown that experimental inoculation of *Nocardia asteroides* or *Nocardia transvalensis* in 10-day-old cockerels resulted in depression, gasping and emaciation [39]. *Aerococcus viridans* has been isolated in meat chickens with hepatitis [40] and *Corynebacteria* may cause diphtheria [41]. *Brachybacterium, Brevibacterium* and *Jeotgalicoccus* which were present mostly in low-performing farms at higher levels require further investigation for their role in causing dysbiosis or subclinical infection in meat chickens. In humans, *Brevibacterium* spp was considered as an opportunistic cause of blood and cardiovascular infection in the immunocompromised humans [42], *Brachybacterium nesterenkovii* has been isolated in the blood stream infections and root canal infection in humans [43, 44], so they may have some negative consequences when colonizing chickens. It is important to note that the detected taxa are dependent on relative abundances since the measurement and analysis is compositional by nature. This means that non-relevant changes in taxa abundances will also change the relative abundance of the identified taxon [45]. Thus, it is advisable to further evaluate the identified taxon related to high and low-performing farms more thoroughly via quantitative PCR in future studies.

In conclusion, the study identified microbial taxa present in poultry dust and excreta and their association with high and low-performance at flock-level. Such easily collected samples are a useful research tool for evaluating microbiota composition longitudinally in commercial flocks to find microbial signatures that correlate with flock health and productivity status. Examining multiple production cycles from the same farms and additional integrator companies is necessary to evaluate the reproducibility of the findings in this study. If the specific bacterial taxa associate with flock productive performance would be reproducible, this would assist in the development of a PCR panel for specific microbiota to evaluate and perhaps predict flock productive performance or in the evaluation of management intervention targeting gut health.

## Supplementary Information

**Additional file 1.** Details of meat chicken farms used in this study. Farms A1 to A4 belong to company A and Farms B1 to B4 belong to company B.

**Additional file 2.** Permutational multivariate analysis of variance (PERMANOVA) results showing the influence of sample type, company, age, performance and their interaction on the bacterial community structure. Df = degrees of freedom, R² = proportion of the variance explained by each model, PERMDISP = permutational analyses of multivariate dispersion.

**Additional file 3.** Principal-coordinate analysis plot using Bray-Curtis dissimilarity showing variation in the bacterial community structure by farm performance (high vs low), bird age (7, 14, 21, 28 and 35), sample type (dust and excreta) and company (A and B).

**Additional file 4.** Taxonomic assignment of the bacteria at phylum level in dust and excreta stratified by company.

**Additional file 5.** Bacteria taxa (genus level) that are shared between dust and excreta and unique to dust and excreta in company A farms.

**Additional file 6.** Bacteria taxa (genus level) that are shared between dust and excreta and unique to dust and excreta in company B farms.

**Additional file 7.** Distinguishing taxa between dust and excreta stratified by company. Linear discriminant analysis effect size was performed on the top 50 most abundant bacterial taxa (genus level) across all ages.

**Additional file 8.** Bacteria taxa (genus level) that are shared between company A and B farms and UNIQUE to company A and B farms in dust.

**Additional file 9.** Bacteria taxa (genus level) that are shared between company A and B farms and UNIQUE to company A and B farms in excreta.

**Additional file 10.** Distinguishing taxa between companies (A and B) stratified by sample type (dust and excreta). Linear discriminant analysis effect size was performed on the top 50 most abundant bacterial taxa (genus level) across all ages.

**Additional file 11.** Genera that were significantly different between high and low-performance farms of company A in dust samples. The results are based on differences of mean abundance tested with Wilcoxon rank-sum test. P-values are corrected with false discovery rate (q-value).

**Additional file 12.** Genera that were significantly different between high and low-performance farms of company B in dust samples. The results are based on differences of mean abundance tested with Wilcoxon rank-sum test. P-values are corrected with false discovery rate (q-value).

**Additional file 13.** Genera that were significantly different between high and low-performance farms of company A in pooled excreta samples. The results are based on differences of mean abundance tested with Wilcoxon ranksum test. P-values are corrected with false discovery rate (q-value).

**Additional file 14.** Genera that were significantly different between high and low-performance farms of company B in pooled excreta samples. The results are based on differences of mean abundance tested with Wilcoxon ranksum test. P-values are corrected with false discovery rate (q-value).

## Data Availability

All the data related to this article were presented inside the manuscript and supplementary files. The sequence data used for analysis is available in NCBI under BioProject accession number PRJNA730489.

## Abbreviations

AUC: Area under curve
DNA: Deoxyribonucleic acid
PCoA: Principal-coordinate analysis
PERMDISP: Permutational analyses of multivariate dispersions
PERMANOVA: Permutational multivariate analysis of variance
16S rRNA: 16S ribosomal ribonucleic acid
