# Supplementary material for: Microbial taxa in dust and excreta associated with the productive performance of commercial meat chicken flocks
Source: Anim Microbiome. 2021 Oct 2;3:66. doi: 10.1186/s42523-021-00127-y (PMC8487525; doi:10.1186/s42523-021-00127-y)
Supplement: Supplementary file 1 — Additional file 1. Details of meat chicken farms used in this study. Farms A1 to A4 belong to company A and Farms B1 to B4 belong to company B. [file 42523_2021_127_MOESM1_ESM.docx]

**Additional file 1.** Details of meat chicken farms used in this study. Farms A1 to A4 belong to company A and Farms B1 to B4 belong to Company B.

| **Farm** | **System type** | **N. poultry houses** | **N. birds placed/ house** | **Ventilation type** ^a^ | **Housing type** | **Bedding material** | **Litter re-use** | **Water source** | **Productive performance (Quartile)**^c^ | | **Flock issues** ^d^ |
| --- | --- | --- | --- | --- | --- | --- | --- | --- | --- | --- | --- |
| **A1** | Conventional | 6 | 44,500 | Artificial | Curtain-sided | Wood shavings | No | Bore | | High (Q2) | - |
| **A2** | Conventional | 5 | 14,500 | Natural | Curtain-sided | Wood shavings | Yes | Surface | | Low (Q4) | Wet litter |
| **A3** | Conventional | 2 | 25,500 | Artificial | Curtain-sided | Sawdust | No | Town | | Low (Q4) | - |
| **A4** | Conventional | 5 | 21,000 | Natural + Artificial ventilation ^b^ | Curtain-sided | Biobedding | Yes | Surface | | High (Q2) | Wet litter |
| **B1** | Conventional | 11 | 41,400 | Artificial | Fully enclosed | Straw | No | Surface | | Low (Q3) | - |
| **B2** | Free-range | 6 | 40,000 | Artificial | Fully enclosed | Straw | No | Bore | | High (Q1) | - |
| **B3** | Free-range | 6 | 41,400 | Artificial | Fully enclosed | Straw | No | Surface | | Low (Q4) | - |
| **B4** | Conventional | 10 | 41,000 | Artificial | Fully enclosed | Straw | No | Surface | | High (Q1) | - |

^a^ Artificial – tunnel fan-forced ventilation

^b^ Out of 2 poultry houses of farm A4, one had natural ventilation and another had artificial ventilation

^c^ Productive performance of the studied batch of birds at the end of the production cycle within each company. Quartile (Q) indicates the farm ranking within the company, Q1 = performance within the best 25% farms; Q2 = between 25-50%; Q3 = between 50-75%; Q4 = bottom 25%.

^d^ - = no issue reported.
