# Supplementary material for: Microbial taxa in dust and excreta associated with the productive performance of commercial meat chicken flocks
Source: Anim Microbiome. 2021 Oct 2;3:66. doi: 10.1186/s42523-021-00127-y (PMC8487525; doi:10.1186/s42523-021-00127-y)
Supplement: Supplementary file 2 — Additional file 2. Permutational multivariate analysis of variance (PERMANOVA) results showing the influence of sample type, company, age, performance and their interaction on the bacterial community structure. Df = degrees of freedom, R² = proportion of the variance explained by each model, PERMDISP = permutational analyses of multivariate dispersion. [file 42523_2021_127_MOESM2_ESM.docx]

**Additional file 2.** Permutational multivariate analysis of variance (**PERMANOVA**) results showing the influence of sample type, company, age, performance and their interaction on the bacterial community structure. Df = degrees of freedom, R² = proportion of the variance explained by each model, PERMDISP = permutational analyses of multivariate dispersion.

| **Source** | **df** | **Sum of squares** | **Pseudo-F** | **R^2^** | **P-value** | **PERMDISP** |
| --- | --- | --- | --- | --- | --- | --- |
| Company | 1 | 81457 | 48.46 | 0.06 | 0.001 | <0.001 |
| Age | 4 | 67022 | 9.97 | 0.05 | 0.001 | <0.001 |
| Sample type | 1 | 149370 | 88.87 | 0.12 | 0.001 | <0.001 |
| Performance | 1 | 12742 | 7.58 | 0.01 | 0.001 | 0.07 |
| Company x Age | 4 | 25798 | 3.84 | 0.02 | 0.001 |  |
| Company x Sample type | 1 | 63895 | 38.02 | 0.05 | 0.001 |  |
| Company x Performance | 1 | 8258 | 4.91 | 0.01 | 0.001 |  |
| Age x Sample type | 4 | 28449 | 4.23 | 0.02 | 0.001 |  |
| Age x Performance | 4 | 11771 | 1.75 | 0.01 | 0.001 |  |
| Sample type x Performance | 1 | 6632 | 3.95 | 0.01 | 0.001 |  |
| Company x Age x Sample type | 4 | 18725 | 2.79 | 0.01 | 0.001 |  |
| Company x Age x Performance | 4 | 14118 | 2.10 | 0.01 | 0.001 |  |
| Company x Sample type x Performance | 1 | 5971 | 3.55 | 0.005 | 0.001 |  |
| Age x Sample type x Performance | 4 | 10026 | 1.49 | 0.01 | 0.001 |  |
| Company x Age x Sample type x Performance | 4 | 13751 | 2.05 | 0.01 | 0.001 |  |
| Res | 349 |  |  |  |  |  |
