# Supplementary material for: Microbial taxa in dust and excreta associated with the productive performance of commercial meat chicken flocks
Source: Anim Microbiome. 2021 Oct 2;3:66. doi: 10.1186/s42523-021-00127-y (PMC8487525; doi:10.1186/s42523-021-00127-y)
Supplement: Supplementary file 3 — Additional file 3. Principal-coordinate analysis plot using Bray-Curtis dissimilarity showing variation in the bacterial community structure by farm performance (high vs low), bird age (7, 14, 21, 28 and 35), sample type (dust and excreta) and company (A and B). [file 42523_2021_127_MOESM3_ESM.docx]

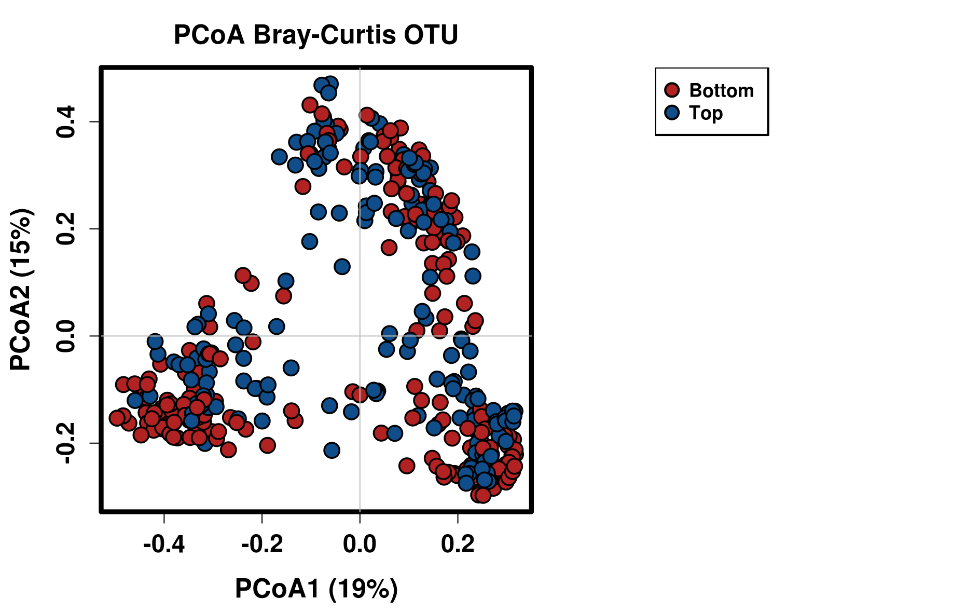

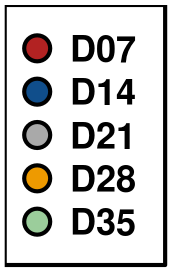

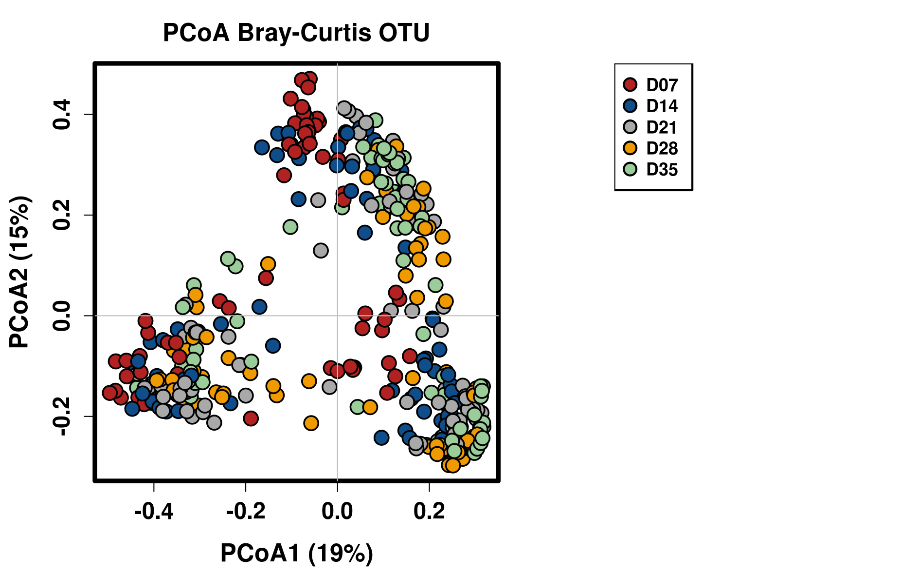

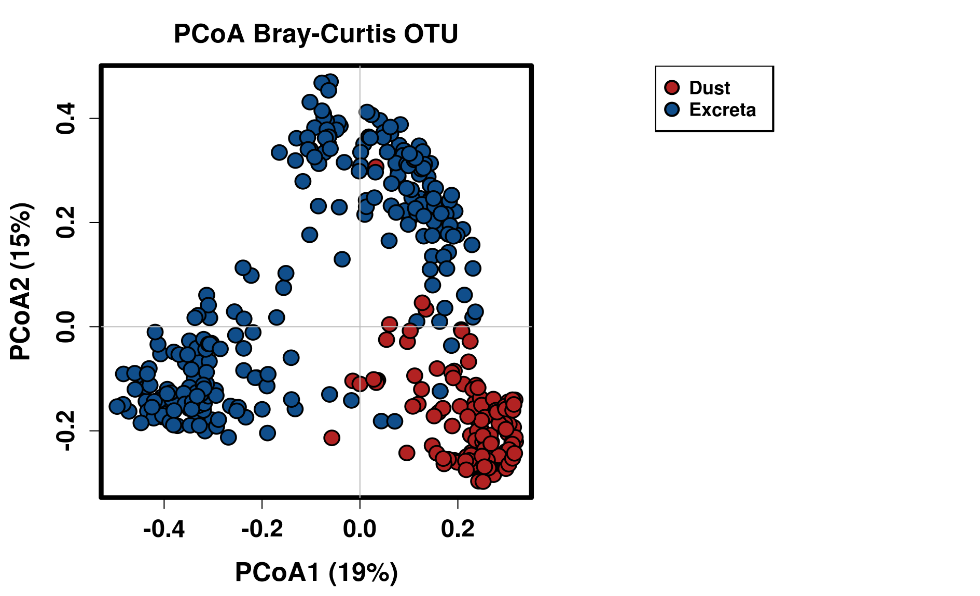
**
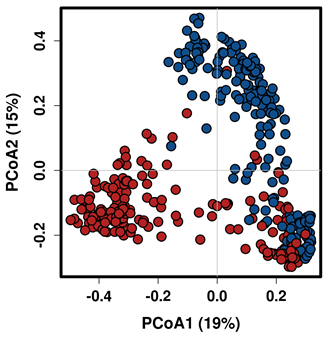
**


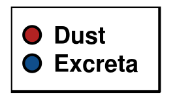

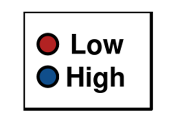

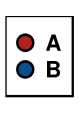


Performance

Bird age (days)

Sample type

Company

**Additional file 3.** Principal-coordinate analysis plot using Bray-Curtis dissimilarity showing variation in the bacterial community structure by farm performance (high vs low), bird age (7, 14, 21, 28 and 35), sample type (dust and excreta) and company (A and B).
