# Supplementary material for: Microbial taxa in dust and excreta associated with the productive performance of commercial meat chicken flocks
Source: Anim Microbiome. 2021 Oct 2;3:66. doi: 10.1186/s42523-021-00127-y (PMC8487525; doi:10.1186/s42523-021-00127-y)
Supplement: Supplementary file 4 — Additional file 4. Taxonomic assignment of the bacteria at phylum level in dust and excreta stratified by company. [file 42523_2021_127_MOESM4_ESM.docx]

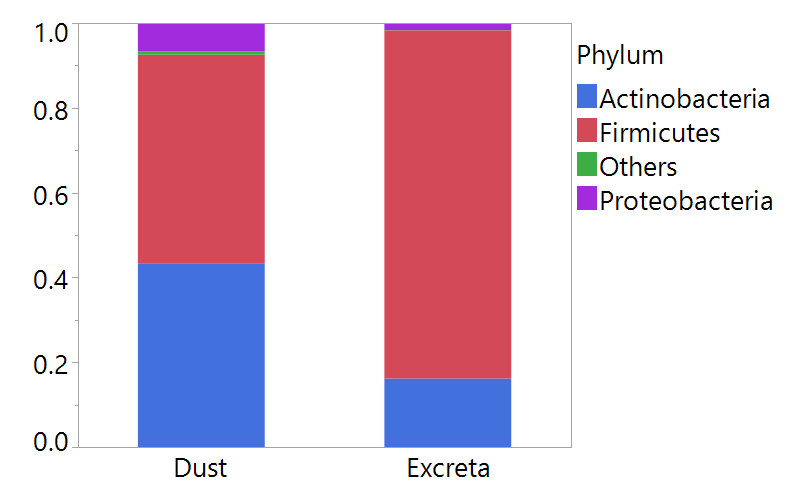

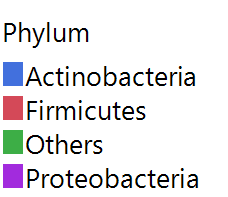

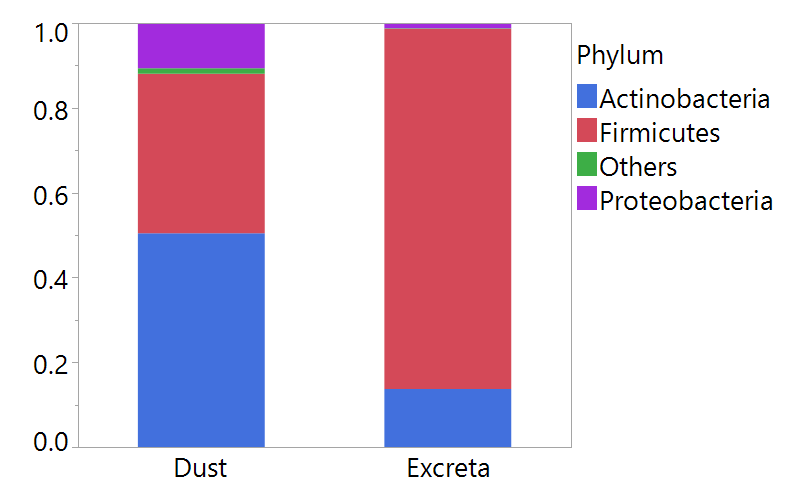


Relative abundance (%)

Relative abundance (%)

Company A

Company B

**Additional file 4.** Taxonomic assignment of the bacteria at phylum level in dust and excreta stratified by company.
