# Supplementary material for: Microbial taxa in dust and excreta associated with the productive performance of commercial meat chicken flocks
Source: Anim Microbiome. 2021 Oct 2;3:66. doi: 10.1186/s42523-021-00127-y (PMC8487525; doi:10.1186/s42523-021-00127-y)
Supplement: Supplementary file 7 — Additional file 7. Distinguishing taxa between dust and excreta stratified by company. Linear discriminant analysis effect size was performed on the top 50 most abundant bacterial taxa (genus level) across all ages. [file 42523_2021_127_MOESM7_ESM.docx]

**
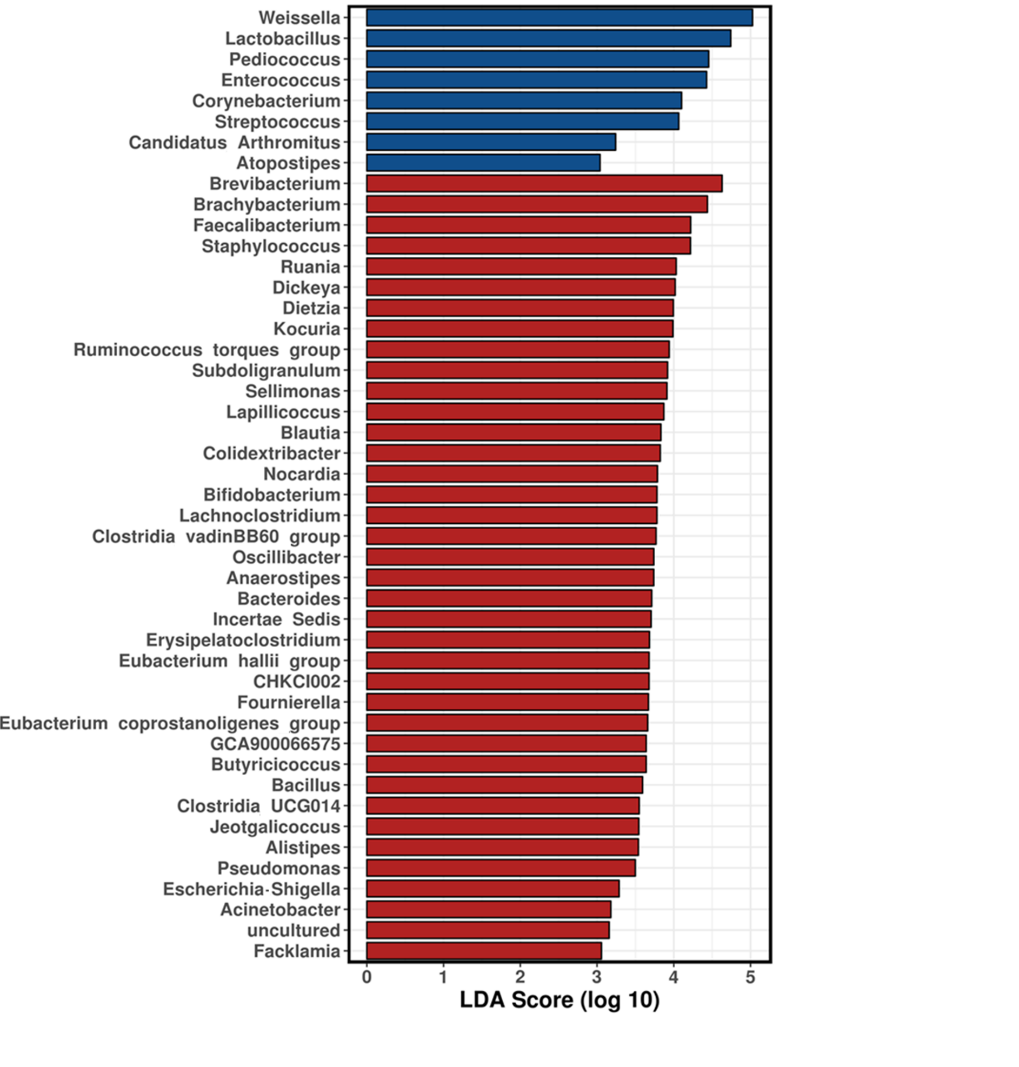
**
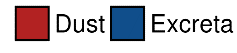

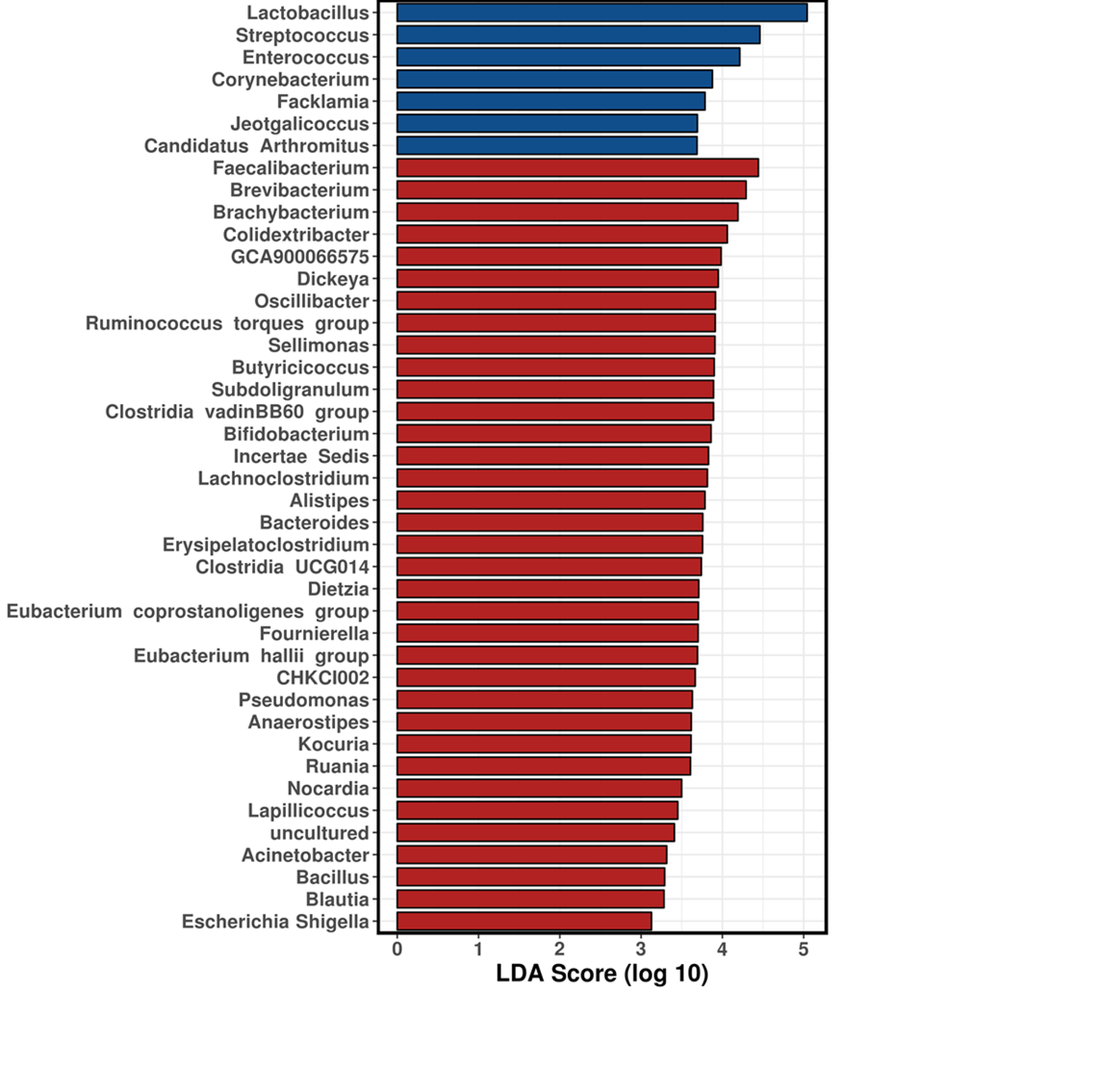

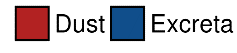
50 **Company A Company B**

**Additional file 7.** Distinguishing taxa between dust and excreta stratified by company. Linear discriminant analysis effect size was performed on the top 50 most abundant bacterial taxa (genus level) across all ages.
