# Supplementary material for: Microbial taxa in dust and excreta associated with the productive performance of commercial meat chicken flocks
Source: Anim Microbiome. 2021 Oct 2;3:66. doi: 10.1186/s42523-021-00127-y (PMC8487525; doi:10.1186/s42523-021-00127-y)
Supplement: Supplementary file 10 — Additional file 10. Distinguishing taxa between companies (A and B) stratified by sample type (dust and excreta). Linear discriminant analysis effect size was performed on the top 50 most abundant bacterial taxa (genus level) across all ages. [file 42523_2021_127_MOESM10_ESM.docx]

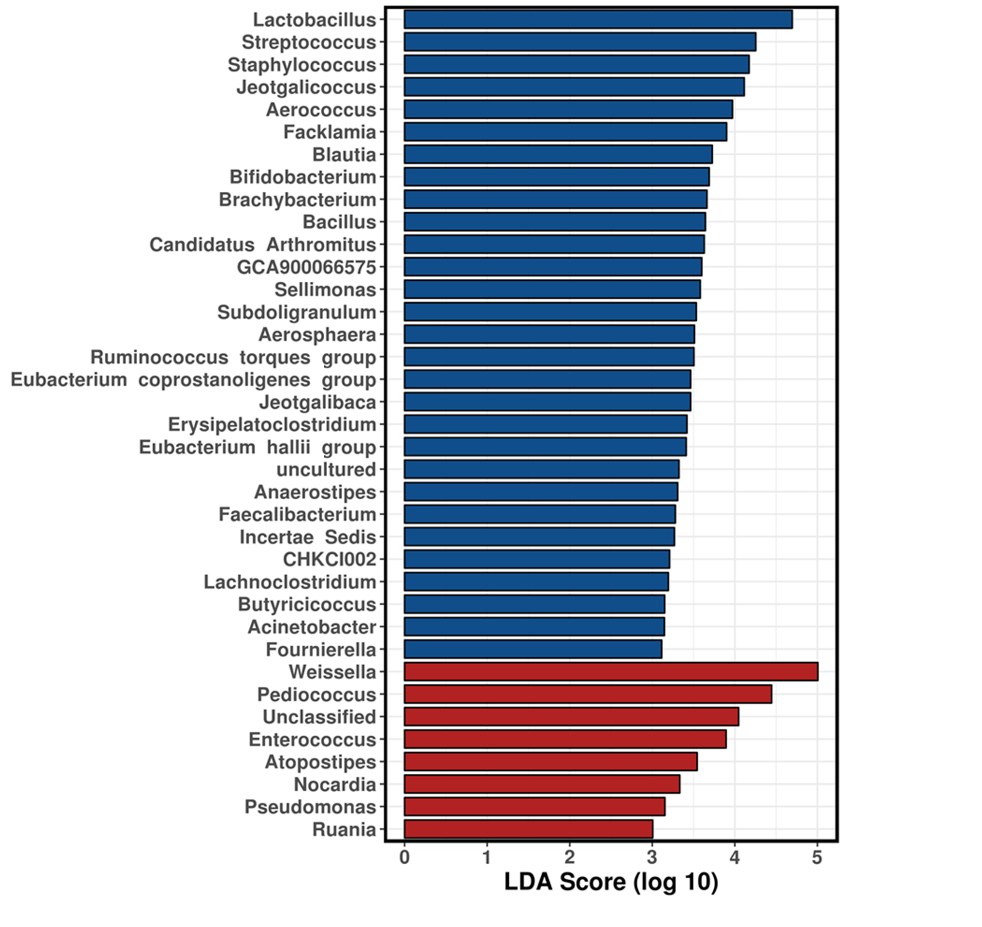

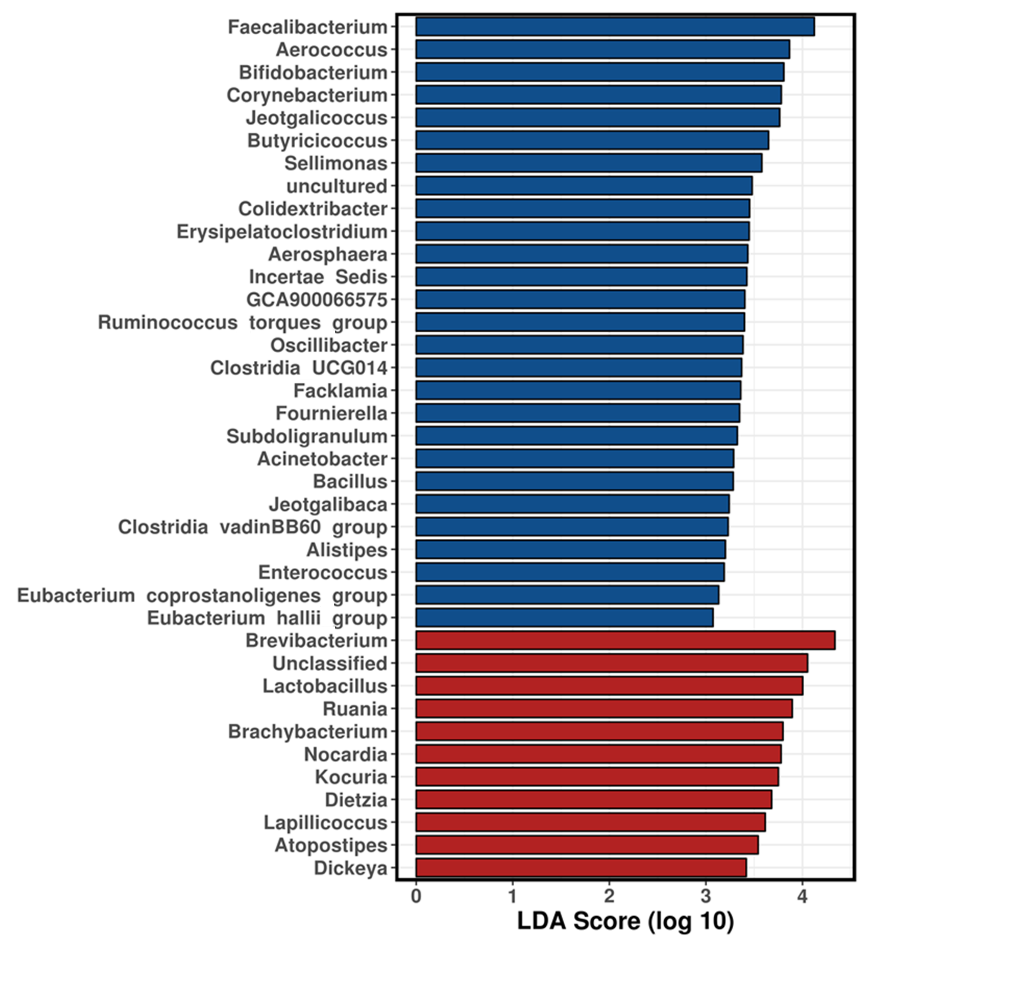
5

**Dust**


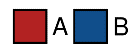


**Excreta**


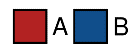


**Additional file 10.** Distinguishing taxa between companies (A and B) stratified by sample type (dust and excreta). Linear discriminant analysis effect size was performed on the top 50 most abundant bacterial taxa (genus level) across all ages.
