# Supplementary material for: Microbial taxa in dust and excreta associated with the productive performance of commercial meat chicken flocks
Source: Anim Microbiome. 2021 Oct 2;3:66. doi: 10.1186/s42523-021-00127-y (PMC8487525; doi:10.1186/s42523-021-00127-y)
Supplement: Supplementary file 11 — Additional file 11. Genera that were significantly different between high and low-performance farms of company A in dust samples. The results are based on differences of mean abundance tested with Wilcoxon rank-sum test. P-values are corrected with false discovery rate (q-value). [file 42523_2021_127_MOESM11_ESM.docx]

**Additional file 11.** Genera that were significantly different between high and low-performance farms of company A in dust samples. The results are based on differences of mean abundance tested with Wilcoxon rank-sum test. P-values are corrected with false discovery rate (q-value).

| **Age of birds (days)** | **Taxa** | **q-value** | **Fold change** | **Low-performing farm [abundance sqrt(TSS)]** | **High-performing farm [abundance sqrt(TSS)]** |
| --- | --- | --- | --- | --- | --- |
| Day 21 | *Brevibacterium* | 0.004 | -1.42 | 5.62 | 3.95 |
|  | *Dietzia* | 0.004 | -1.87 | 1.53 | 0.82 |
|  | *Ruania* | 0.02 | -2.16 | 1.45 | 0.67 |
|  | *Brachybacterium* | 0.02 | -1.43 | 4.58 | 3.21 |
|  | *Lapillicoccus* | 0.02 | -3.08 | 0.80 | 0.26 |
|  | *Nocardia* | 0.04 | -2.23 | 0.94 | 0.42 |
|  |  |  |  |  |  |
| Day 28 | *Ruania* | 0.008 | -1.66 | 1.11 | 0.67 |
