# Supplementary material for: Microbial taxa in dust and excreta associated with the productive performance of commercial meat chicken flocks
Source: Anim Microbiome. 2021 Oct 2;3:66. doi: 10.1186/s42523-021-00127-y (PMC8487525; doi:10.1186/s42523-021-00127-y)
Supplement: Supplementary file 12 — Additional file 12. Genera that were significantly different between high and low-performance farms of company B in dust samples. The results are based on differences of mean abundance tested with Wilcoxon rank-sum test. P-values are corrected with false discovery rate (q-value). [file 42523_2021_127_MOESM12_ESM.docx]

**Additional file 12.** Genera that were significantly different between high and low-performance farms of company B in dust samples. The results are based on differences of mean abundance tested with Wilcoxon rank-sum test. P-values are corrected with false discovery rate (q-value).

| **Age of birds (days)** | **Taxa** | **q-value** | **Fold change** | **Low-performing farms [abundance sqrt (TSS)]** | **High-performing farms [abundance sqrt (TSS)]** |
| --- | --- | --- | --- | --- | --- |
|  | *Dickeya* | 0.01 | 1.34 | 0.82 | 1.10 |
| Day 35 | *Lapillicoccus* | 0.01 | -8.00 | 0.40 | 0.05 |
|  | *Ruania* | 0.01 | -5.22 | 0.47 | 0.09 |
|  | *Weissella* | 0.02 | 2.79 | 0.28 | 0.78 |
