# Supplementary material for: Microbial taxa in dust and excreta associated with the productive performance of commercial meat chicken flocks
Source: Anim Microbiome. 2021 Oct 2;3:66. doi: 10.1186/s42523-021-00127-y (PMC8487525; doi:10.1186/s42523-021-00127-y)
Supplement: Supplementary file 13 — Additional file 13. Genera that were significantly different between high and low-performance farms of company A in pooled excreta samples. The results are based on differences of mean abundance tested with Wilcoxon ranksum test. P-values are corrected with false discovery rate (q-value). [file 42523_2021_127_MOESM13_ESM.docx]

**Additional file 13.** Genera that were significantly different between high and low-performance farms of company A in pooled excreta samples. The results are based on differences of mean abundance tested with Wilcoxon rank-sum test. P-values are corrected with false discovery rate (q-value).

| **Age of birds (days)** | **Taxa** | **q-value** | **Fold change** | **Low-performing farm [abundance sqrt (TSS)]** | **High-performing farm [abundance sqrt (TSS)]** |
| --- | --- | --- | --- | --- | --- |
| Day 7 | *Enterococcus* | 0.003 | 2.07 | 1.50 | 3.11 |
|  | Unclassified | 0.009 | 2.64 | 2.07 | 5.47 |
|  | *Nocardia* | 0.009 | -12.00 | 0.24 | 0.02 |
|  | *Corynebacterium* | 0.01 | -2.46 | 1.01 | 0.41 |
|  | *Pediococcus* | 0.01 | -2.86 | 3.38 | 1.18 |
|  | *Dietzia* | 0.02 | -6.00 | 0.36 | 0.06 |
|  | *Escherichia-Shigella* | 0.04 | 3.14 | 0.22 | 0.69 |
|  |  |  |  |  |  |
| Day 28 | *Pediococcus* | 0.04 | -3.08 | 1.48 | 0.48 |
