# Supplementary material for: Microbial taxa in dust and excreta associated with the productive performance of commercial meat chicken flocks
Source: Anim Microbiome. 2021 Oct 2;3:66. doi: 10.1186/s42523-021-00127-y (PMC8487525; doi:10.1186/s42523-021-00127-y)
Supplement: Supplementary file 14 — Additional file 14. Genera that were significantly different between high and low-performance farms of company B in pooled excreta samples. The results are based on differences of mean abundance tested with Wilcoxon ranksum test. P-values are corrected with false discovery rate (q-value). [file 42523_2021_127_MOESM14_ESM.docx]

**Additional file 14.** Genera that were significantly different between high and low-performance farms of company B in pooled excreta samples. The results are based on differences of mean abundance tested with Wilcoxon rank-sum test. P-values are corrected with false discovery rate (q-value).

| **Age of birds (days)** | **Taxa** | **q-value** | **Fold change** | **Low performing farms [abundance sqrt (TSS)]** | **High performing farms [abundance sqrt (TSS)]** |
| --- | --- | --- | --- | --- | --- |
| Day 14 | *Jeotgalicoccus* | 0.004 | -3.84 | 1.42 | 0.37 |
|  | *Corynebacterium* | 0.005 | -2.90 | 3.39 | 1.17 |
|  | *Aerococcus* | 0.005 | -3.63 | 2.18 | 0.60 |
|  | *Candidatus Arthromitus* | 0.008 | 3.59 | 0.27 | 0.97 |
|  | *Facklamia* | 0.01 | -8.13 | 0.65 | 0.08 |
|  | *Brachybacterium* | 0.01 | -3.71 | 1.15 | 0.31 |
|  | *Brevibacterium* | 0.01 | -3.38 | 0.71 | 0.21 |
|  | *Aerosphaera* | 0.03 | -15 | 0.45 | 0.03 |
|  | *Jeotgalibaca* | 0.03 | Inf | 0.31 | 0.00 |
|  | *Staphylococcus* | 0.04 | -1.71 | 3.47 | 2.03 |
|  | *Acinetobacter* | 0.047 | -14.5 | 0.29 | 0.02 |
| Day 28 | *Faecalibacterium* | 0.045 | 4.38 | 0.08 | 0.35 |
|  | *Facklamia* | 0.045 | -2.1 | 1.40 | 0.65 |
|  | *Subdoligranulum* | 0.045 | -2.38 | 0.16 | 0.38 |
|  | *Streptococcus* | 0.045 | 2.04 | 0.97 | 1.98 |
|  | Unclassified | 0.045 | -1.37 | 1.73 | 1.26 |
|  | *Jeotgalicoccus* | 0.045 | -1.46 | 1.99 | 1.36 |
|  | *Aerosphaera* | 0.045 | -5.42 | 0.38 | 0.07 |
|  | *Erysipelatoclostridium* | 0.045 | 3.00 | 0.03 | 0.09 |
| Day 35 | *Enterococcus* | 0.002 | 4.76 | 0.21 | 1.00 |
